# Comparative Evaluation of Hydrogel Dip-Coating on Cone and Pyramid Microneedle Arrays Fabricated by LCD 3D Printing

**DOI:** 10.3390/pharmaceutics18050518

**Published:** 2026-04-24

**Authors:** Feria Hasanpour, Oliwia Kordyl, Zuzanna Styrna, Barbara Jadach, Tomasz Osmałek, Ferhan Ayaydin, Mária Budai-Szűcs, Anita Kovács, Szilvia Berkó

**Affiliations:** 1 Institute of Pharmaceutical Technology and Regulatory Affairs, Faculty of Pharmacy, University of Szeged, 6 Eötvös Str., H-6720 Szeged, Hungarybudai-szucs.maria@szte.hu (M.B.-S.);; 2 Chair and Department of Pharmaceutical Technology, 3D Printing Division, Poznan University of Medical Sciences, 3 Rokietnicka Str., 60-806 Poznań, Polandtosmalek@ump.edu.pl (T.O.); 3 Student’s Research Group of Pharmaceutical Technology, The Student Scientific Society of Poznan University of Medical Sciences, 5 Rokietnicka Str., 60-806 Poznań, Poland; 4 Functional Cell Biology and Immunology Advanced Core Facility (FCBI), Hungarian Centre of Excellence for Molecular Medicine (HCEMM), University of Szeged, 6 Korányi Ave., H-6720 Szeged, Hungary; 5 Agribiotechnology and Precision Breeding for Food Security National Laboratory, Institute of Plant Biology, HUN-REN Biological Research Centre, 62 Temesvári Blvd., H-6726 Szeged, Hungary

**Keywords:** additive manufacturing, microneedle arrays, 3D printing, lidocaine, dermal delivery, hydrogel coating, conical and pyramidal geometry

## Abstract

**Background**: Additive manufacturing provides a rapid and flexible alternative to conventional micromolding for producing microneedle systems. This study evaluates the potential of a cost-effective LCD 3D printer for fabricating microneedle arrays (MNAs) and investigates how the geometry of MNAs and the formulation of hydrogel influence the performance of lidocaine-coated arrays. **Methods**: Conical and pyramidal MNAs, along with a reservoir plate, were designed and manufactured. Lidocaine-loaded and placebo hydrogels with two different polymer concentrations were prepared for dip-coating using both single and multilayer applications. Mechanical resistance and insertion efficiency were evaluated under controlled compression. The physicochemical behavior of the hydrogels were characterized, including pH, spreadability, adhesiveness, and rheological behavior. The uniformity of the coating was analyzed using 3D confocal microscopy. Drug loading was quantified by HPLC, drug release was studied using Franz diffusion cells, and skin penetration was confirmed by 3D confocal imaging and Raman mapping. **Results**: Conical microneedles exhibited high mechanical integrity, showing only a 2% reduction in height compared to 4% for pyramidal MNAs. Stronger drug signals were achieved in deeper skin layers with the conical geometry, indicating enhanced penetration, while pyramidal MNAs provided slightly higher lidocaine loading due to their larger lateral surface. Hydrogels with higher polymer content produced more stable, uniform coatings, particularly when applied in three layers. Rapid drug release was observed, with over 70% of the drug delivered within minutes. **Conclusions**: LCD 3D printing offers a cost-effective approach for fabricating MNAs with suitable structural stability and sharpness. The optimized hydrogel formulation ensured uniform coverage, as well as maximal and consistence penetration, making this platform a promising candidate for the dermal delivery of other potent drugs.

## 1. Introduction

Dermal drug delivery enables localized and targeted administration of therapeutic agents directly to the skin, thereby maximizing local pharmacological efficacy while substantially minimizing systemic exposure [1]. It is widely used in dermatology, pain management, and as a topical anesthetic prior to minor surgical and cosmetic interventions. In contrast, transdermal drug delivery facilitates the passage of active compounds across the skin and into the systemic circulation, providing a controlled and sustained release, circumventing first-pass hepatic metabolism, and offering a potent, noninvasive alternative to conventional parenteral injection [2,3,4,5].

Recent advances in microneedle technology have successfully integrated these two concepts, enabling either localized or systemic delivery as a function of microneedle material composition, structural design parameters (including needle height, base dimensions, and interneedle spacing), and penetration depth [6,7].

This dual functionality positions microneedles as a versatile delivery platform, providing rapid drug release comparable to conventional hypodermic injections, while retaining the safety profile and user-friendliness associated with analogous topical formulations [8].

Microneedle arrays (MNAs) can be manufactured from a diverse range of materials, including polymers and inorganic substrates, unlike traditional hypodermic needles, which are typically made from stainless steel. This flexibility in both material selection and design allows for precise control over the geometry of MNA, enabling the customization of the microneedle architecture to be tailored to specific therapeutic indications [3,9].

Recent advances in additive manufacturing and three-dimensional (3D) printing technologies—including material extrusion or fused deposition modeling (FDM), powder bed fusion (PBF), and vat photopolymerization (VPP)—have substantially broadened the design space and fabrication capabilities of microneedle (MN)-based systems. Among these techniques, VPP is particularly advantageous for MN fabrication owing to its high spatial resolution, smooth surface finish, and excellent geometric fidelity, which collectively enable precise control over microneedle architecture and the integration of functional features. VPP encompasses several methodological variants, such as stereolithography (SLA), digital light processing (DLP), continuous liquid interface production (CLIP), and liquid crystal display (LCD) printing. Compared to other VPP modalities and additive manufacturing approaches, LCD printing presents several distinct advantages for MN fabrication. Specifically, it is more cost-effective than SLA and DLP, while affording higher resolution than FDM [10,11,12,13,14,15,16]. Although two-photon polymerization (2PP) can achieve superior feature resolution and dimensional accuracy, its widespread implementation is constrained by elevated equipment costs and slow production rates. CLIP-based processes similarly offer increased fabrication speed but remain less accessible due to higher capital and operating expenditures [17,18,19,20] (Table 1).

MNAs can be broadly categorized into several types based on their structural configuration and associated drug-release mechanisms. Solid microneedles, employed in the “poke and patch” approach, create transient microchannels in the stratum corneum, facilitating the subsequent diffusion of topically applied pharmacological agents. Coated MNAs, used in the “coat and poke” method, carry the drug on their external surface. These coatings dissolve rapidly upon insertion into the skin, rendering them particularly suitable for rapid-onset interventions such as local anesthesia. Dissolving MNAs, based on the “poke and release” strategy, are composed of biodegradable polymers that encapsulate the drug within the needle matrix and release it as the microneedles dissolve, enabling single-step, residue-free delivery. Hollow MNAs, corresponding to the “poke and flow” technique, function as miniaturized hypodermic syringes, allowing controlled infusion of liquid formulations into deeper dermal layers and supporting the delivery of larger doses or systemic delivery [33,34,35,36]. Finally, hydrogel-forming MNAs swell upon insertion, forming an in situ reservoir that supports sustained drug release or enables interstitial fluid extraction for diagnostic sampling [37].

For localized dermal therapies, coated MNAs have demonstrated remarkable versatility, capable of delivering a broad spectrum of active compounds, including small molecules, proteins, DNA, viruses, and nanoparticles at doses spanning from nanograms to hundreds of micrograms. This allows precise and rapid administration directly into the skin [35,36,38]. Their performance critically depends on the uniformity and spatial selectivity of the coating process, as efficient delivery is achieved only when drug deposition is confined to the microneedle shafts, thereby minimizing wastage on the backing substrate. Conventional immersion, spray, and layer-by-layer coating techniques generally yield lower drug loading compared to dip-coating or inkjet-based methods. Since the amount of drug released from the microneedles ultimately defines the dose delivered to the skin, achieving highly uniform coatings is particularly crucial for potent agents that require precise and accurate doses [36,38,39,40].

Among the available methodologies, dip-coating has demonstrated particular effectiveness, as it enables precise regulation of coating thickness and deposition through systematic optimization of key process parameters, including formulation viscosity, immersion time, withdrawal speed, and the number of dipping cycles. In this technique, pre-fabricated solid or hydrogel MNAs are repeatedly immersed in a drug-containing formulation composed of film-forming polymers and water-soluble excipients, followed by a controlled drying step to produce a uniform, coherent, and strongly adherent coating along the microneedle shafts [36,40].

Hydrogel-based dip-coatings, particularly those incorporating hydroxyethylcellulose, have been demonstrated to enhance drug stability, inhibit aggregation, and preserve therapeutic efficacy. These coatings form a biocompatible film matrix that stabilizes the active pharmaceutical ingredient during storage and administration, and subsequently releases it into the epidermal and dermal layers following cutaneous insertion. This approach improves local bioavailability, enables controlled release, and renders hydrogel-coated MNAs highly suitable for both rapid and sustained transdermal drug delivery [41].

Lidocaine, one of the most extensively used local anesthetics, is frequently employed as a model compound for evaluating microneedle-based delivery systems. However, the stratum corneum, characterized by its densely packed structure and relatively low water content (15–20%), constitutes a major barrier to dermal drug penetration [19,42]. Despite the relatively low molecular weight of lidocaine (~0.234 kDa), passive diffusion from conventional topical formulations is limited, necessitating prolonged application periods to achieve adequate anesthesia [4,43]. Hydrogel-coated MNAs overcome this limitation by generating transient microchannels across the stratum corneum, thereby enabling precise, rapid, and efficient intradermal delivery of lidocaine. This approach supports a faster onset of action, improved dermal deposition, reduced required doses, and improved patient comfort [40,44,45,46].

While previous works have largely focused on high-resolution techniques such as SLA, this study investigates LCD-based 3D-printed MNAs coated with lidocaine-loaded hydrogel. The LCD approach was selected due to its balanced resolution (18–50 μm), low cost, and suitability for rapid prototyping [12]. We systematically compare conical and pyramidal microneedle geometries in terms of insertion efficiency, mechanical strength, coating uniformity, drug loading, release behavior, and skin permeation performance. In parallel, we evaluate the influence of hydrogel formulation and coating strategy, including polymer concentration and single versus multilayer dip-coating, on coating quality.

## 2. Materials and Methods

### 2.1. Materials

Lidocaine was obtained from Hungaropharma Ltd. (Budapest, Hungary). The transparent and UV-curable resin (Anycubic^®^ Standard Clear, batch number RPTT02418C0301) was supplied by Anycubic Corporation (Shenzhen, China). Carbopol^®^ EZ-3 (batch no. 0102679988) was sourced from Lubrizol Corp. (Brussels, Belgium). Glycerol (85% *w*/*w*, batch no. 094004) was acquired from Fagron (Olomouc, Czech Republic), while triisopropanolamine (batch no. MKCM7547) was purchased from Sigma-Aldrich (St. Louis, MO, USA). Furthermore, analytical-grade solvents and reagents were procured from Avantor Performance Materials (Gliwice, Poland), including ethanol (99.8%, batch no. 1317-03-24), isopropanol (batch no. 0808/10/15), acetonitrile (batch no. 1090-09-23), and potassium dihydrogen phosphate (batch no. 0008/01/22). High-purity water was generated on-site using the Simplicity^®^ purification system from Merck Millipore (Billerica, MA, USA). Human skin was acquired from a Caucasian female patient who underwent an abdominal plastic surgery at the University of Szeged, Department of Dermatology and Allergology. The investigations were performed with the approval of the Hungarian Medical Research Council (ETT-TUKEB, registration number: BMEÜ/2339-3/2022/EKU).

### 2.2. Methods

#### 2.2.1. Design of Microneedle Arrays

**Figure 1:**
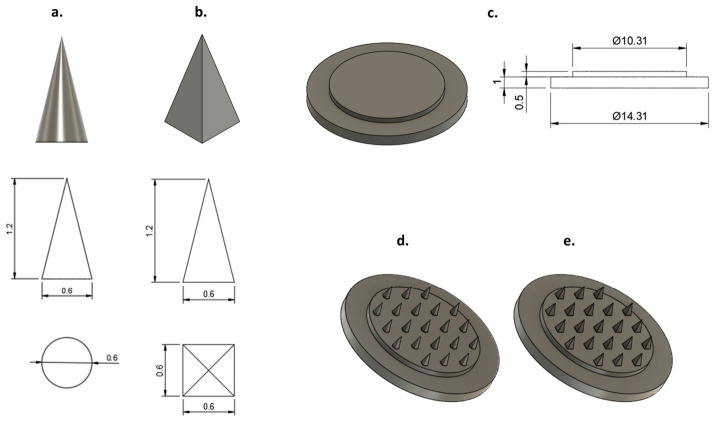
CAD models (top) and corresponding two-dimensional (2D) engineering drawings (bottom) of a single microneedle with two distinct geometries: (**a**) a conical configuration and (**b**) a pyramidal configuration with a square base. (**c**) CAD representation (left) and associated 2D technical drawings (right) of the double-layered support base. The finalized CAD assemblies of the 21-microneedle conical array (**d**) and the 21-microneedle pyramidal array (**e**) are also presented.

The MNAs were designed using AUTODESK^®^ FUSION 360^®^ (version 2601.0.90; Autodesk Inc., San Rafael, CA, USA). The resulting CAD models were exported in STL format and subsequently processed with Photon Workshop V3.3.0 (Anycubic^®^, Shenzhen, China) for slicing and conversion to PM3/G-code, thereby enabling accurate, layer-by-layer additive manufacturing. Two distinct microneedle geometries were developed: a conical configuration (Figure 1a) and a pyramidal configuration with a square base (Figure 1b). Both designs shared identical dimensions, comprising a needle height of 1200 μm, a base width of 600 µm, and an interneedle spacing of 2000 µm, parameters selected to reduce skin insertion resistance and enhance penetration efficiency. Each array consisted of 21 uniformly distributed microneedles mounted on a double-layered base platform to improve mechanical robustness. The base structure consisted of a lower layer (diameter 14.31 mm, height 1 mm) and an upper layer (diameter 13.31 mm, height 0.5 mm) (Figure 1c). Consequently, the finalized CAD models contained two microneedle array designs: conical (Figure 1d) and pyramidal (Figure 1e).

To enhance MN insertion performance and account for the elastic and deformable characteristics of the skin, a theoretical approach was developed to correlate the required insertion force with MN interspacing. This relationship was further supported by finite element modeling and experimental validation. The analysis demonstrated that increasing the interneedle spacing decreases the insertion force, thereby mitigating the “bed-of-nails effect” and promoting more uniform penetration with lower variability [47]. Furthermore, the MN aspect ratio was identified as another critical design parameter, with higher aspect ratios (3:1–4:1) corresponding to sharper needle tips and greater insertion depth [13,48]. An aspect ratio of 2:1 was selected to maximize the surface area available for coating and drug loading by allowing a broader base without increasing the array density [7]. Additionally, an interneedle spacing of 2000 μm was chosen to decrease insertion resistance and alleviate the bed-of-nails effect, thereby enhancing the uniformity and reliability of skin penetration.

#### 2.2.2. Fabrication of Microneedle Arrays

The MNAs were fabricated using LCD-based 3D printing with a photocurable resin (Anycubic^®^ Standard Clear Resin) on an Anycubic Photon Mono M5 3D printer, equipped with a 10.1-inch LCD screen offering a resolution of 12K and a printing accuracy of up to 19 µm (Anycubic Technology, Hong Kong, China). The printer was operated at a layer thickness (Z resolution) of 0.03 mm, enabling the production of microstructures with high dimensional accuracy and well-defined tip geometry. Fabrication was conducted at a 45° printing orientation, with an exposure (curing) time of 7.5 s per layer.

**Figure 2:**
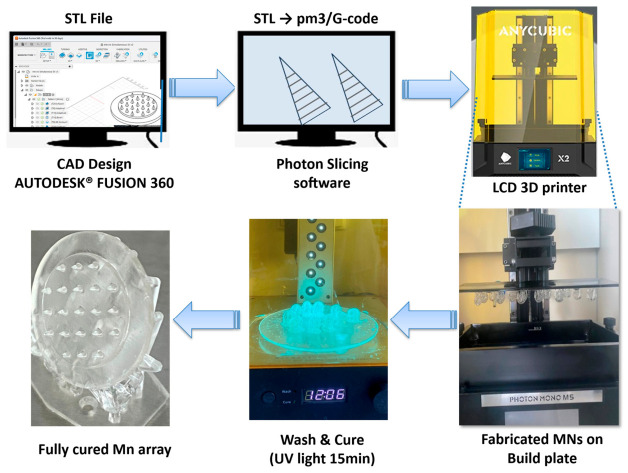
Schematic representation of the MNA fabrication workflow, illustrating the progression from the initial CAD model through the sequential manufacturing and processing stages to the formation of the final microneedle structures, immediately prior to their release from the underlying support structures.

Following printing, unpolymerized resin was removed by washing the constructs with isopropanol using a Wash and Cure Machine (Anycubic^®^ Technology, Hong Kong, China). Subsequently, the arrays were subjected to ultraviolet (UV) irradiation (385–405 nm) for 15 min in the same unit to ensure complete curing and structural stabilization via photopolymerization, as depicted in the microneedle fabrication workflow (Figure 2).

The biocompatibility of this resin system has been previously evaluated under similar fabrication conditions, where Microtox^®^ testing indicated no acute toxicity of the cured microneedle structures [49]. However, further cytotoxicity studies are required to fully establish biocompatibility for clinical applications.

#### 2.2.3. Investigation Methods of Microneedle Arrays

##### In Vitro Insertion Efficiency

The insertion performance of both microneedle geometries was assessed in vitro using a TA.XT Plus Texture Analyzer (Stable Micro Systems Ltd., Surrey, UK) and eight uniformly cut Parafilm^®^ M squares (2 × 2 cm) as a synthetic skin simulant. The films were stacked to achieve a total thickness of approximately 1.016 mm (with an individual layer thickness of ~127 µm). The MNAs were affixed to the movable arm of a Texture Analyzer with double-sided adhesive tape, while the Parafilm^®^ M stack was positioned on a foam-cushioned metal base to provide a more compliant support.

During testing, the probe was lowered at a speed of 0.05 mm/s until a target force of 30 N was reached. The force condition was selected based on reported manual thumb-applied pressure conditions [50]. The force was maintained for 30 s and the probe was subsequently withdrawn at 1 mm/s.

Each experimental condition was performed in triplicate. Following insertion, the Parafilm^®^ M layers were carefully separated and examined to determine the number of layers penetrated by the microneedles, enabling comparative analysis of insertion depth and mechanical performance between the two geometries. Insertion efficiency was further quantified by counting the number of perforations in each layer and calculating the fraction of pierced sites according to Equation (1) [49,51,52,53].

(1) $$\%holes=\frac{The\;number\;of\;holes\;observed}{The\;number\;of\;microneedles}\times 100$$

##### Mechanical Properties

The mechanical properties of the MNAs were evaluated using a TA.XT Plus Texture Analyzer (Stable Micro Systems Ltd., Surrey, UK) equipped with a cylindrical probe of 10 mm in diameter. The MNAs were affixed to the probe and compressed against a metallic base at a crosshead speed of 0.50 mm/s, with a compressive load of 30 N applied for 30 s to simulate mechanical stress conditions, and subsequently unloaded at a speed of 1 mm/s.

The post-compression MN tip morphology was examined with a light microscope LEICA DM6 B (Leica Microsystems GmbH, Wetzlar, Germany) and a Leica DMM205-FA stereo-zoom microscope (Leica Microsystems, Heidelberg, Germany), combining LED-ring epi-illumination with transmitted light illumination. All images were acquired under identical illumination and zoom settings to ensure comparability.

The microneedle height was measured before (H1) and after (H2) the compression test, and the percentage height reduction was calculated according to Equation (2) [54]:

(2) $$Variation\;in\;needle\;height\left(\%\right)=\frac{H1-H2}{H1}\times 100\%$$

#### 2.2.4. Preparation and Characterization of Hydrogel for Coating

##### Preparation of Hydrogels

Hydrogels containing varying concentrations of Carbopol^®^ EZ-3 polymer were prepared by initially mixing 85% glycerol with deionized water in a plastic vessel. Carbopol^®^ EZ-3 was then gradually dispersed over the surface of the liquid phase, and the dispersion was stirred at 600 rpm for 30 min using a mechanical stirrer (CAT R 50, CAT M. Zipperer GmbH, Ballrechten Dottingen, Germany). In a separate step, 2% (*w*/*w*) lidocaine was dissolved in 96% (*v*/*v*) ethanol, and the resulting solution was then added to the polymer–glycerol dispersion under continuous stirring. Placebo hydrogels were prepared following the same procedure, but without lidocaine. Gelation was induced by the dropwise addition of a triisopropanolamine solution to adjust the pH. In lidocaine-containing formulations, the intrinsic alkalinity of the drug was sufficient to induce gelation, thereby eliminating the need for an additional neutralizing agent. The qualitative and quantitative compositions of the resulting gels are presented in Table 2.

##### Measurement of pH

The pH of each hydrogel formulation (5 g) was measured at ambient temperature using a Testo 206 pH meter equipped with a pH2 probe (Testo SE & Co. KGaA, Lenzkirch, Germany). All measurements were performed in triplicate to ensure the accuracy and reproducibility of the data.

##### Spreadability and Adhesiveness

The spreadability and adhesiveness of hydrogel formulations intended for microneedle coating were assessed using a TA.XT Plus Texture Analyzer (Stable Micro Systems Ltd., Surrey, UK) equipped with a 5 kg load cell. A cylindrical probe (10 mm diameter) was affixed to the microneedle array and aligned above the hydrogel surface. Measurements were conducted in compression mode, in which the probe was lowered at a pre-test speed of 0.20 mm/s until a trigger force of 0.002 N indicated contact with the sample. Thereafter, the hydrogel was compressed to a depth of 2.0 mm at the same speed, after which the probe was withdrawn at a post-test speed of 0.20 mm/s. All experiments were performed in triplicate.

##### Rheological Analysis

Rheological measurements were performed to characterize the structural integrity, consistency, and mechanical behavior of the gel formulations, as well as to evaluate the impact of lidocaine incorporation. Experiments were conducted using a Physica MCR 302 Modular Compact Rheometer (Anton Paar GmbH, Graz, Austria) equipped with a cone–plate measuring system (CP25; diameter 25 mm, cone angle 1°, gap 0.05 mm).

The flow behavior was assessed by applying a controlled shear rate (γ), increasing from 0.1 to 100 s^−1^ (upward curve) and afterwards decreasing from 100 to 0.1 s^−1^ (downward curve) over a total duration of 300 s. Viscosity (η) was continuously monitored as a function of shear rate, and the apparent viscosity at 50 s^−1^ was selected for comparative analysis among samples [55].

Viscoelastic properties were evaluated using oscillatory rheological tests. Initially, an amplitude sweep was conducted to determine the linear viscoelastic region (LVER). Following this, a frequency sweep within the LVER was performed over an angular frequency (ω) range of 0.1–100 rad·s^−1^ to obtain the storage modulus (G′) and loss modulus (G″). All measurements were carried out in triplicate at 25 °C.

#### 2.2.5. Coating Method for Microneedle Systems

**Figure 3:**
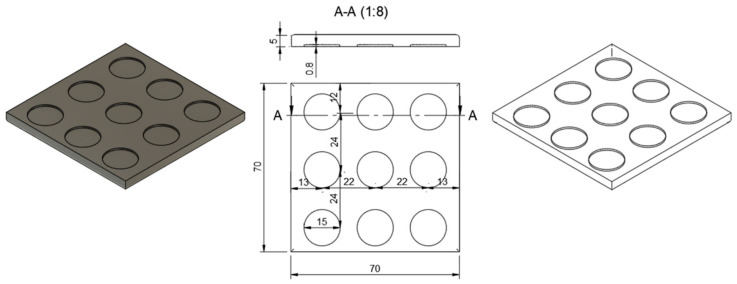
CAD model (**left**) and corresponding 2D technical drawing (**right**) of the coating plate.

The microneedle arrays were coated with Hydrogel 1, Hydrogel 2 or blank formulations using a thin-film dip-coating method [36]. A custom reservoir plate was fabricated using the same LCD-based 3D printer employed for microneedle production, utilizing a photocurable resin (Anycubic^®^ Standard Clear Resin) on an Anycubic^®^ Photon Mono 5 system (Anycubic Technology, Hong Kong, China) under the manufacturer’s standard printing parameters. The plate was designed as a 70 × 70 mm square substrate with a thickness of 5 mm and featured nine circular reservoirs (15 mm in diameter, 0.8 mm in depth) arranged in a 3 × 3 grid. The spatial configuration of these reservoirs was aligned with the microneedle array geometry to ensure a coating coverage of two-thirds of the microneedle length (Figure 3, section A–A) [49].

Semi-manual dip-coating was carried out using a TA.XT Plus Texture Analyzer (Stable Micro Systems Ltd., Surrey, UK) equipped with a cylindrical probe of 10 mm diameter. Microneedle arrays (MNAs) were mounted on the probe and immersed into reservoirs containing the hydrogel formulations at an insertion speed of 0.50 mm/s, until a penetration depth of 0.600 mm and a trigger force of 5 N were reached. The probe was then maintained at the target position for 5 s, with a post-speed setting of 0.50 mm/s. After completion of the coating step, the probe was retracted at a speed of 1 mm/s. MNAs were prepared with either a single-layer or a triple-layer coating. For the triple-layer coating, the dipping procedure was repeated three times, with each coating cycle separated by an 8-h drying period.

#### 2.2.6. Investigation of Coated Microneedle Systems

##### Microscopic Analysis of Microneedle Arrays

The hydrogel coating procedure and the ex vivo skin penetration performance of the microneedles were investigated using a Leica DMI8–Stellaris 5 laser scanning confocal microscope (Leica Microsystems, Heidelberg, Germany). Two distinct microneedle geometries were coated with a rhodamine B-loaded hydrogel using either a single-layer or a triple-layer coating protocol, allowing comparison of coating uniformity and adhesion, as well as evaluation of the effectiveness of multilayer application along the microneedle shafts.

Prior to skin insertion, confocal z-stack images of the coated microneedle arrays were acquired to visualize and compare the spatial distribution and thickness of the coatings generated by the single- and triple-layer methods at corresponding positions along the needle height. Subsequently, the MNAs were inserted into excised, full-thickness human skin for 15 min and then withdrawn. Penetration depth was quantified by detecting rhodamine B-containing hydrogel residues within the skin tissue. Rhodamine B served as a fluorescent tracer, allowing differentiation of the hydrogel coating from the intrinsic autofluorescence of the skin and enabling precise visualization of the spatial distribution and depth of coating penetration at different magnifications [56].

Rhodamine B fluorescence was detected using excitation with a 526 nm laser, and the emitted signal was collected over an emission range of 555–718 nm. Autofluorescence originating from the 3D-printed microneedle material was recorded using a 405 nm laser for excitation and an emission range of 421–492 nm, enabling distinct visualization of the needle structures. Skin autofluorescence was excited at 448 nm and detected within an emission window of 456–516 nm. All autofluorescence signals were displayed using green pseudocoloring to enhance image contrast. Images were acquired using 5× HCPL Fluotar objectives (numerical aperture, N.A. 0.15) and 10× HCPL Fluotar objectives (N.A. 0.3) with Leica Application Suite X software (version 4.9). Multiple z-sections were combined to visualize 3D reconstructions using the Leica 3D software plugin (version 4.9). Identical microscope settings were used for all images included in the intensity comparison.

##### Analysis of Drug Content

For the quantitative determination of drug content, individual MNAs were transferred into glass vials containing 10 mL of phosphate-buffered saline (PBS, pH 7.4) and incubated for 2 h on a rotary mixer to ensure complete dissolution of the loaded lidocaine. The resulting solutions were subsequently analyzed using a validated high-performance liquid chromatography (HPLC) method on a Shimadzu Nexera X2 UHPLC system (Shimadzu, Kyoto, Japan) equipped with LabSolution Lite software (version 5.82) and a reversed-phase C18 column (ZORBAX Eclipse XDB-C18, 4.6 × 150 mm, 5 µm; Phenomenex, Torrance, CA, USA).

The mobile phase consisted of 0.1% (*v*/*v*) phosphoric acid in water (solvent A) and acetonitrile (solvent B), run in gradient mode starting at a 90:10 ratio (A:B, *v*/*v*), which was linearly adjusted to 40:60 over 6 min, and then returned to the initial composition between 6.1 and 10 min. The flow rate was maintained at 0.8 mL/min, and both the column and autosampler temperatures were controlled at 25 °C. Lidocaine was monitored at a detection wavelength of 230 nm with an injection volume of 5 µL, yielding a retention time of approximately 4.2 min. All measurements were conducted in triplicate to ensure analytical precision and reproducibility.

##### In Vitro Release Test (IVRT)

Drug release was investigated using Franz diffusion cells (Phoenix RDS automatic diffusion system, Teledyne LABS, Thousand Oaks, CA, USA). The receptor compartment was maintained at 32 ± 0.5 °C to simulate physiological skin temperature and was continuously stirred at 200 rpm. Phosphate-buffered saline (PBS), with pH 7.4, was employed as the receptor medium to ensure sink conditions. MNAs were positioned directly onto the diffusion cell orifice in the absence of a membrane, permitting direct contact with the receptor medium [49]. At predefined time points (6, 12, 30, 45 min; 1 and 2 h), 0.3 mL aliquots of receptor fluid were withdrawn from the Franz diffusion cells, transferred into HPLC vials, and immediately replaced with equal volumes of fresh buffer to maintain a constant diffusion volume. Sample analysis was performed by HPLC as described in Section Analysis of Drug Content. All experiments were conducted in triplicate.

##### Ex Vivo Skin Permeation Study

Full-thickness human skin samples were prepared for Raman mapping by placing them on filter paper saturated with PBS and excising 4 cm^2^ sections for analysis. The samples were then punctured with MNAs coated with three layers of Hydrogel 1, or its corresponding blank formulation, using a TA.XT Plus Texture Analyzer (Stable Micro Systems Ltd., Surrey, UK) as previously described (Section Mechanical Properties), except that the metallic plate was replaced by the human skin sample. Following a 1-h treatment period, the skin specimens were cut into strips and cryosectioned using a Leica CM1950 Cryostat (Leica Biosystems GmbH, Wetzlar, Germany) to obtain 15 µm transverse sections, which were subsequently mounted on aluminum-coated slides for Raman spectroscopic analysis. Reference spectra of lidocaine and Hydrogel 1 (with and without lidocaine) were acquired using a Thermo Fisher DXR Dispersive Raman Spectrometer (Thermo Fisher Scientific Inc., Waltham, MA, USA) equipped with a CCD camera and a 780 nm laser (exposure time 6 s; 24 scans per measurement; cosmic ray and fluorescence corrections applied; laser power 10 mW; slit width: 25 µm).

Subsequently, Raman mapping was conducted to determine the spatial distribution of lidocaine within the skin cross-sections. A 780 nm excitation laser was employed at a power of 24 mW [57,58]. Using a 50× objective lens, a 100 × 500 µm area was scanned with a step size of 50 µm in both the X and Y directions, acquiring 24 spectra at each measurement position. Untreated skin samples served as the negative control. Data acquisition and spectral processing were carried out using OMNIC™ 8.2 software for dispersive Raman spectroscopy (Thermo Fisher Scientific Inc., Waltham, MA, USA). Spectral profiling was performed by comparing the acquired spectra with reference spectra of the hydrogel-lidocaine formulation within the 200–1500 cm^−1^ wavenumber range, which was selected to isolate characteristic vibrational bands of lidocaine, minimize overlap with hydrogel-associated spectral features, and thereby facilitate robust and reproducible Raman mapping [58].

#### 2.2.7. Statistical Analysis

Statistical analyses were conducted using Prism for Windows, version 5.0 (GraphPad Software Inc., La Jolla, CA, USA). Differences between groups were evaluated by two-way analysis of variance (ANOVA), followed by Bonferroni’s post hoc test. For the mechanical testing data, differences were considered statistically significant at *p* ≤ 0.01 (**) compared with the control group. In the in vitro release test (IVRT), no statistically significant differences were observed at any of the measured time points [59].

## 3. Results and Discussion

### 3.1. Characterization of Microneedle Arrays

#### 3.1.1. In Vitro Insertion Efficiency

**Figure 4:**
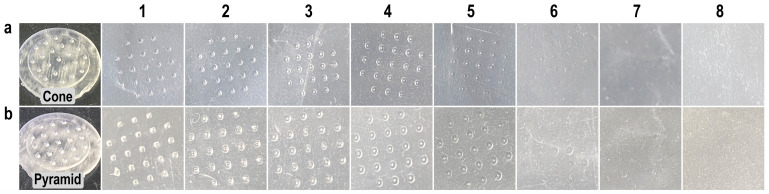
Representative images of perforations in Parafilm^®^ M layers caused by MNs: (**a**) conical and (**b**) pyramidal geometries. Layers 1–8 correspond sequentially to the stacked Parafilm^®^ M sheets. The interneedle distance for both MNAs is 2 mm.

**Figure 5:**
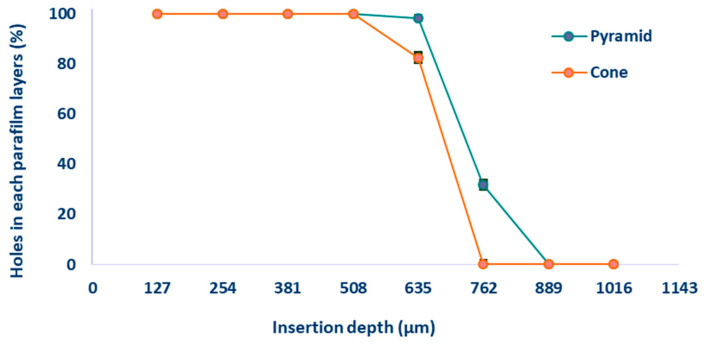
Quantitative assessment of MN penetration through eight sequential layers of Parafilm^®^ M (individual layer thickness: ~127 µm), reported as the percentage of needles successfully perforating each layer for the two distinct geometrical designs. Data are expressed as mean ± SD (*n* = 3).

Parafilm^®^ M has been widely adopted as a model material for replicating the mechanical properties of human skin [50,51]. Figure 4a,b demonstrate the insertion performance of conical and pyramidal MNAs through stacked Parafilm^®^ M layers. For conical MNAs, complete penetration of all microneedles was achieved up to the fourth layer (cumulative thickness: 508 µm), with only partial perforations observed in the fifth layer. In contrast, the pyramidal MNAs attained complete insertion through the fifth layer and produced limited perforations in the sixth layer. No penetration was observed beyond the seventh layer for either MNA design. Afterwards, the number of perforations in each layer was quantified and expressed as a percentage of the total microneedle insertions for the different MNA geometries (Figure 5).

#### 3.1.2. Mechanical Properties

**Figure 6:**
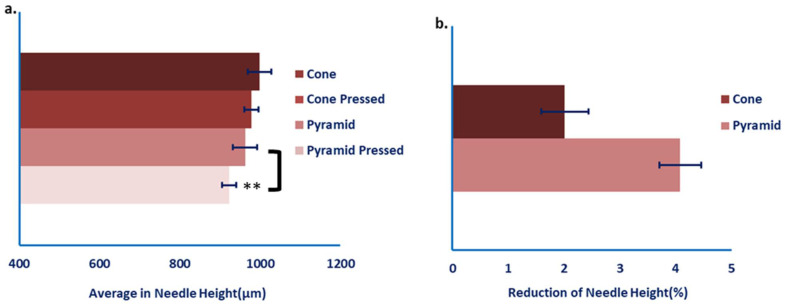
(**a**) Microneedle height quantified relative to the nominally designed geometry and (**b**) percentage reduction in mean microneedle height following compression for both geometrical configurations. Data are presented as mean ± SD (*n* = 10). A statistically significant difference was observed for the pyramid-shaped pressed group compared with the unpressed pyramid-shaped group (*p* ≤ 0.01, **).

Analysis of MN tip morphologies revealed that none of the fabricated structures fully retained the target geometry of 1200 µm in height, indicating dimensional shrinkage due to LCD-based additive manufacturing and subsequent post-processing steps (Figure 6a)**.** Conical microneedles exhibited an average pre-pressing height of 998.7 µm, corresponding to a 16.8% deviation from the nominal design, whereas pyramidal microneedles reached an average height of 962.5 µm, representing a 19.8% reduction.

**Figure 7:**
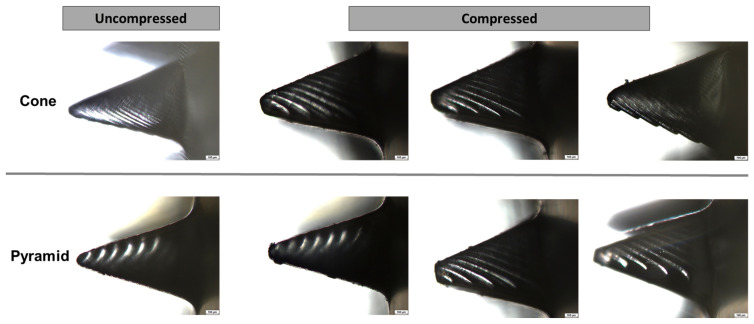
Representative microscopic images of microneedles in the uncompressed (**left**) and compressed (**right**) states. The upper row displays cone-shaped microneedles, whereas the lower row presents pyramid-shaped microneedles.

Following the pressing procedure, all microneedle geometries showed only minor dimensional changes, with no evidence of bending or fracture. However, a measurable decrease in MN height was observed, amounting to a non-significant reduction of 2.02% for conical and a statistically significant reduction of 4.09% for pyramidal microneedles (Figure 6b and Figure 7), indicating differing levels of structural stability between the two geometries. The consistently greater dimensional loss in pyramidal microneedles suggests inferior mechanical robustness compared with the conical configuration.

This disparity is likely attributable to the sharp edges and planar faces of the pyramidal structures, which can act as stress concentrators and facilitate localized deformation, whereas the continuous, smooth profile of conical microneedles promotes a more homogeneous stress distribution during both curing and mechanical loading. Consequently, conical microneedles appear to constitute the most mechanically reliable design for applications that demand reproducible skin penetration performance and high structural stability.

### 3.2. Evaluation of Hydrogels for Coating

#### 3.2.1. Results of pH Measurements

The pH values of the prepared hydrogels ranged between 5.35 and 7.57 (Table 3). For Hydrogel 1, the blank formulation exhibited a pH of 5.65, while the lidocaine-loaded formulation showed an increased pH of 7.27. This suggests that lidocaine incorporation shifted the formulation toward a more basic environment, even in the absence of triisopropanolamine. Since the lidocaine concentration remained constant, the variation in the polymer concentration (lidocaine–polymer ratio) appears to have played a key role in determining the pH of the drug-loaded formulations.

A similar trend was observed for Hydrogel 2, which contained a lower concentration of Carbopol^®^ EZ-3. Upon lidocaine loading, the pH increased to 7.57, while the corresponding blank formulation exhibited a slightly lower pH of 5.35. These findings indicate that Carbopol^®^ EZ-3, a cross-linked poly(acrylic acid) polymer [60,61], contributes to lowering the pH due to its acidic carboxyl functional groups. However, all formulations remained within the acceptable pH range for dermal application.

#### 3.2.2. Results of Spreadability and Adhesiveness

The spreadability and adhesive characteristics of the hydrogels were quantified by analyzing the force–time profiles obtained during texture analysis. Firmness (maximum force) was defined as the peak force recorded during the compression phase, while spreadability was determined as the area under the compression curve. The adhesion force (detachment force) was defined as the maximum negative force detected during probe withdrawal, and adhesiveness was calculated as the area under the negative portion of the curve during the removal phase (Table 4).

Hydrogel 1 exhibited the highest firmness (127.07 ± 5.55 mN) and work of spreading (704.22 ± 58.99 mN·s), compared to Hydrogel 2 (95.58 ± 8.99 mN and 643.55 ± 120.50 mN·s, respectively), indicating a more mechanically robust and structurally stable gel network with an increased resistance to deformation and spreading. Consistently, both adhesion force and adhesiveness were higher for Hydrogel 1 (−55.97 ± 5.22 mN and −692.58 ± 37.45 mN·s, respectively) than for Hydrogel 2 (−49.54 ± 6.04 mN and −590.56 ± 111.21 mN·s). The blank formulations exhibited lower values across all evaluated parameters.

These findings reveal that Hydrogel 1 (containing lidocaine and a higher polymer concentration) possesses superior cohesion and structural integrity during spreading, along with stronger adhesive interactions with the microneedle surface, thereby supporting more durable attachment during dip-coating and subsequent application.

#### 3.2.3. Results of Rheological Characterization

The rheological properties of Hydrogel 1, Hydrogel 2, and their corresponding blank formulations were systematically evaluated to determine their suitability for dip-coating MNs, a critical parameter in achieving a uniform and stable coating. Particular emphasis was placed on flow behavior, viscosity, and viscoelastic characteristics, as these properties govern coating adhesion and retention on the MN surface.

Flow measurements demonstrated that all formulations exhibited non-Newtonian, shear-thinning (pseudoplastic) behavior, with shear stress increasing non-linearly over the applied shear rate range (0–100 s^−1^). Such pseudoplasticity is considered advantageous for dip-coating processes, as it promotes homogeneous gel distribution during immersion while enabling viscosity recovery at rest, thereby improving coating cohesion and adhesion to the substrate.

**Figure 8:**
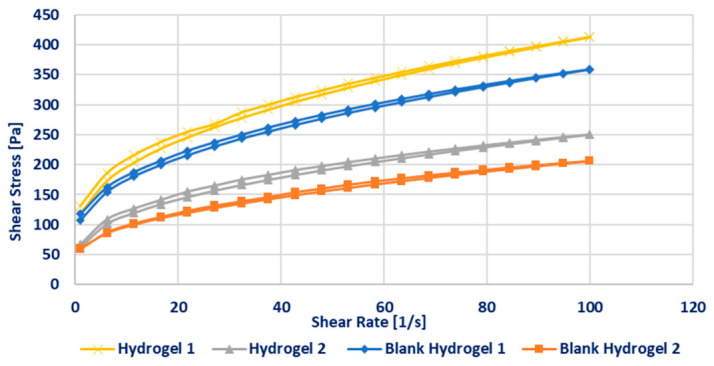
Flow curves of the various hydrogel formulations.

Hydrogel 1 and its corresponding blank formulation displayed a higher yield stress (approximately 100 Pa), nearly twice that of Hydrogel 2 and its blank. This finding indicates a more robust internal network structure and stronger intermolecular interactions within the Formulation 1 series, which can be attributed to its higher polymer content. The elevated yield stress and viscosity provide greater resistance to gravitational forces, facilitating maintenance of the gel layer on the MN surface during and after the dipping process. Additionally, the incorporation of lidocaine further increased gel viscosity, suggesting that the active pharmaceutical ingredient contributes to the structuring of the hydrogel matrix. (Figure 8)**.**

The viscosities measured at a shear rate of 50 s^−1^ fell within the 3000–7000 mPa·s range considered suitable for effective microneedle (MN) dip-coating (Table 5). Among the investigated formulations, Hydrogel 1 exhibited the highest viscosity (6855 ± 128 mPa·s). In comparison, Blank Hydrogel 1 presented a modestly reduced viscosity (6082 ± 259 mPa·s), whereas Hydrogel 2 and Blank Hydrogel 2 displayed substantially lower viscosities of 4118 ± 17 mPa·s and 3220 ± 28 mPa·s, respectively. The elevated viscosity of Hydrogel 1 is expected to facilitate the formation of thicker and more cohesive coating layers, thereby enhancing post-dip adhesion, which is critical for preserving coating integrity [62].

**Figure 9:**
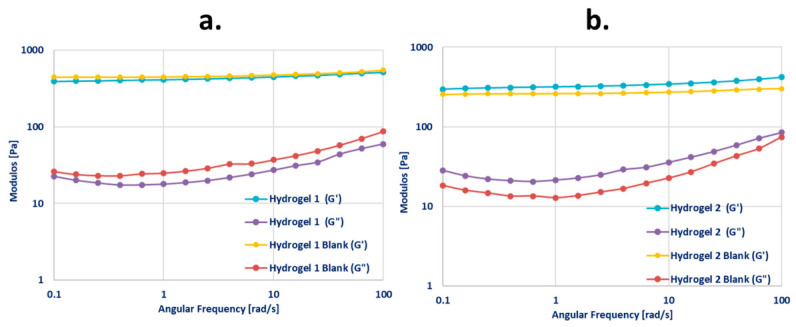
Frequency sweep rheological profiles depicting the storage modulus (G′) and loss modulus (G″) of Hydrogel 1 and its corresponding blank formulation (**a**), and Hydrogel 2 and its respective blank formulation (**b**), as a function of angular frequency.

The frequency sweep data provide insight into the rheological behavior of lidocaine-loaded hydrogels with varying concentrations of Carbopol^®^ and their corresponding drug-free formulations, through analysis of the storage modulus (G′) and loss modulus (G″). Variations in these moduli as a function of angular frequency confirm the viscoelastic character of the systems [63]. All formulations exhibited storage modulus (G′) values consistently exceeding the loss modulus (G″) values, indicating the predominance of elastic, gel-like behavior in both the lidocaine-loaded and blank hydrogels (Figure 9). Hydrogel 1 demonstrated slightly higher G′ and G″ values compared to the Hydrogel 2 formulation, reflecting a more solid-like rheological behavior likely due to its higher polymer content.

Based on the comparative evaluation of the coating hydrogels, Hydrogel 1 was selected for MNA coating due to its optimal rheological and mechanical characteristics, including viscosity, firmness, adhesion, and viscoelasticity. These properties are predictive of forming stable, homogeneous coatings on the microneedle surface and maintaining coating integrity without disruption following insertion.

### 3.3. Evaluation of Coated Microneedle System

#### 3.3.1. Evaluation of the Coating Process

**Figure 10:**
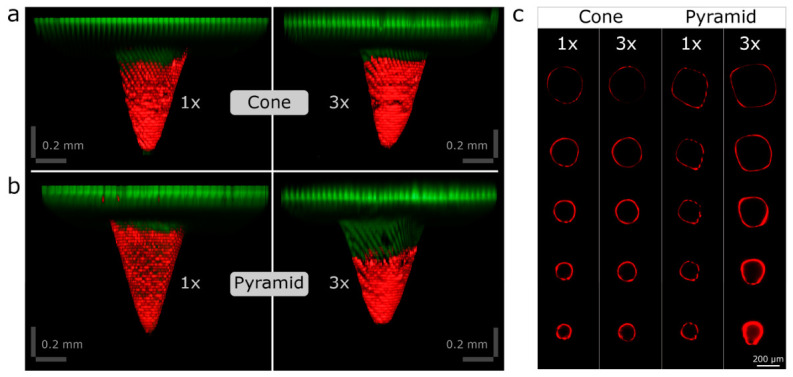
3D confocal microscopy images of Hydrogel 1 (Rhodamine B)-coated MNs. Single-layer (1×) and triple-layer (3×) coatings are presented for cone-shaped (**a**) and pyramid-shaped (**b**) microneedle geometries. (**c**) Optical cross-sectional images depicting the coating thickness at equivalent axial positions along the microneedle shaft for single-layer (1×)- and triple-layer (3×)-coated cone and pyramid microneedles. Rhodamine B fluorescence is shown in red, and the microneedle autofluorescence under violet laser excitation is shown in green.

To gain a more comprehensive understanding of the MNA coating process and to assess the influence of Hydrogel 1, premixed with rhodamine B (used as a fluorescent tracer for visualization), on the microneedle surface, three-dimensional confocal laser scanning microscopy was conducted. Figure 10 presents the two microneedle geometries following (a) a single-layer coating and (b) a triple-layer coating. The single-layer application generated a thin yet continuous surface film, whereas the triple-layer application produced a thicker, more uniform and smoother coating. In both conditions, the coating extended to more than approximately one-half to two-thirds of the total microneedle height, consistent with the intended design specifications. All microneedles exhibited a homogeneous and uniform coating along their entire geometry using the semi-manual dip-coating method. This approach facilitated more consistent and reproducible drug loading compared to the fully manual coating technique.

In addition, confocal 3D microscopy was employed to characterize the spatial distribution of the coating on the microneedles following one- and three-layer applications of Hydrogel 1, using optical cross-sectioning of the needle structures (Figure 10c). The reconstructed 3D images revealed distinct differences in surface coverage in the base, middle, and tip regions. Quantitative analysis indicated a marked increase in coating thickness and associated fluorescence intensity in the pyramidal geometries with successive coating layers, confirming a more homogeneous hydrogel deposition on the microneedles subjected to the three-layer coating protocol.

For subsequent analyses, MNAs with a triple-layer coating and conical or pyramidal geometries were selected.

#### 3.3.2. Investigation of Penetration Efficiency into the Skin

**Figure 11:**
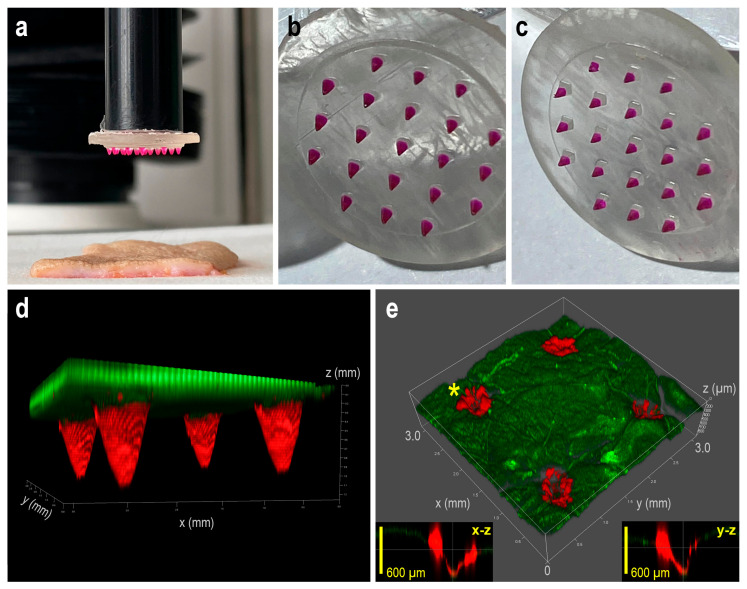
(**a**) Photo of the setup using a texture analyzer to insert coated MNAs into excised full-thickness human skin. (**b**,**c**) Representative macroscopic images of the cone- and pyramid-shaped MNAs coated with rhodamine B-hydrogel, respectively. (**d**) 3D confocal microscopy reconstruction of a segment of MNA coated with rhodamine B-hydrogel (red), with green autofluorescence indicating uncoated regions of the base material; the distance between needle tips is 2 mm. (**e**) 3D reconstruction of a skin section after insertion and removal of the coated MNA. Needle-delivered rhodamine B (red) is visible at four insertion sites as depressed regions along the needle tracks, whereas skin autofluorescence is shown in green. Orthogonal X–Z and Y–Z projections of the z-stack are shown as inset images at the puncture site indicated by an asterisk.

3D confocal laser scanning microscopy was additionally employed to qualitatively assess the penetration efficiency of rhodamine B-hydrogel-coated MNAs in fully excised human skin. Figure 11a shows the microneedle insertion process conducted by a texture analyzer, while Figure 11b,c present photographic images of cone- and pyramid-shaped MNAs following coating with the rhodamine B-loaded hydrogel.

Figure 11d,e display 3D reconstructed confocal micrographs of the coated MNAs and the corresponding excised skin sections following microneedle insertion and subsequent removal, with rhodamine B fluorescence visualized in red and intrinsic skin autofluorescence in green. Orthogonal X–Z and Y–Z projections of the z-stack at the puncture sites (marked by asterisks) confirm successful microneedle insertion and intradermal deposition of the hydrogel coating. The 3D renderings were generated from 25 serial confocal optical sections using Leica LAS X 3D, (version 4.9) software. Analysis of the fluorescence intensity profiles indicates a reproducible penetration depth within the skin, while the spatially uniform fluorescence signal supports homogeneous delivery of the hydrogel formulation.

A supplementary 3D animation was prepared to visualize hydrogel deposition along the microneedle insertion tracks, complementing the static confocal images (Supplementary Video S1). This dynamic illustration allows for continuous observation of fluorescence along the pierced tissue, showing penetration depth and hydrogel distribution. Rotation combined with cross-sectional views (X–Z and Y–Z projections) provides a clear understanding of the spatial relationship between coated microneedles and skin layers.

The experimental approaches used in this study, including the Parafilm insertion model and penetration assessment in excised human skin, provide reliable and complementary evaluation of microneedle insertion efficiency and drug delivery performance. However, these models have inherent limitations. They do not fully replicate physiological conditions, such as microcirculation, metabolic activity, and immune responses, which can influence drug distribution and retention. In addition, differences in hydration and mechanical properties compared to in vivo skin may affect microneedle insertion and delivery behavior. Parafilm also does not reproduce the complex biological structure of human skin. Therefore, further in vivo studies are required to fully establish efficacy and safety under physiological conditions.

### 3.4. Evaluation of Lidocaine-Loaded Microneedle Systems

For the preparation of lidocaine-loaded MNAs, Hydrogel 1 containing 2% (*w*/*w*) lidocaine was employed in a three-layer coating process.

#### 3.4.1. Determination of Drug Content

Following dissolution of the hydrogel coating layers in 10 mL of PBS under continuous orbital shaking for 2 h, pyramidal MNAs exhibited a higher drug loading (236.47 ± 11.74 µg) than cone-shaped MNAs (216.47 ± 6.10 µg). This difference can be attributed to the variation in the lateral surface area of the respective microneedle geometries (Table 6).

#### 3.4.2. In Vitro Drug Release

**Figure 12:**
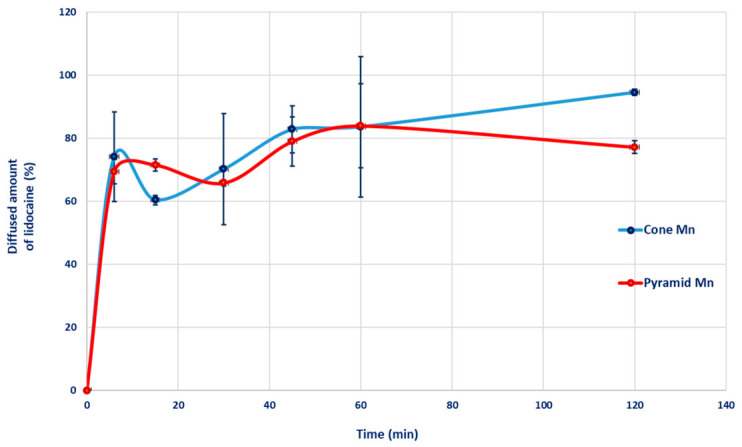
Release profiles of lidocaine from microneedle systems of varying geometries, each coated with Hydrogel 1.

The 2-h drug-release evaluation demonstrated that both microneedle geometries effectively delivered the loaded compound, exhibiting similar release profiles (Figure 12)**.** In both cases, a pronounced initial burst was observed, with approximately 70% of the total drug load being released at the first sampling point, within 6 min. The subsequent non-monotonic release behavior can be attributed to pH-dependent lidocaine ionization and its interactions with the polymer matrix. Carbopol^®^ is a high-molecular-weight poly(acrylic acid) that undergoes dissociation in aqueous media [60]. Lidocaine, as a weak base, undergoes protonation in acidic media. At pH 7.4, approximately 33% of lidocaine is present as the unprotonated free base, whereas about 67% exists in the protonated form [64,65,66]. Electrostatic interactions between protonated lidocaine and Carbopol^®^ lead to the formation of a reversible ion-pair complex [67].

Consequently, a fraction of the lidocaine remains in an unbound state, whereas another fraction is covalently or physically associated with the polymer matrix. The initial burst release can be attributed to rapid depletion of the unbound free-base fraction located near the gel–buffer interface [67]. The following sustained release phase results from the lengthened diffusion path from the interior of the gel [68]. The fluctuating phase occurs when ions from the acceptor phase diffuse into the Carbopol^®^ gel network and electrostatically shield the negatively charged moieties of Carbopol^®^. This charge screening, or partial neutralization, diminishes both intra- and intermolecular electrostatic repulsion, which under normal conditions maintains the anionic Carbopol^®^ chains in an extended conformation and the gel in a highly swollen state. The consequent reduction in repulsive forces can induce a mild gel contraction (syneresis). This contraction expels a portion of the remaining dissolved drug, leading to a secondary, lower-intensity peak in the release profile [67,69].

#### 3.4.3. Ex Vivo Skin Permeation of the Drug

Raman spectroscopy was employed to determine the spatial distribution of the lidocaine-loaded hydrogel within cross sections of full-thickness human skin after pretreatment with conical and pyramidal MNAs. The MNAs were coated with three layers of either the lidocaine-loaded hydrogel or the corresponding placebo formulation and applied for 1 h.

Spectral interpretation was complicated by partial overlap of vibrational bands arising from formulation constituents (lidocaine and hydrogel matrix) with those of endogenous skin components, including lipids, proteins, and nucleic acids [58]. To enhance analytical specificity, data evaluation was restricted to the 200–1500 cm^−1^ wavenumber range, which provided a characteristic spectral fingerprint for the hydrogel–lidocaine system. Microneedles coated with the blank formulation were used as control samples.

**Figure 13:**
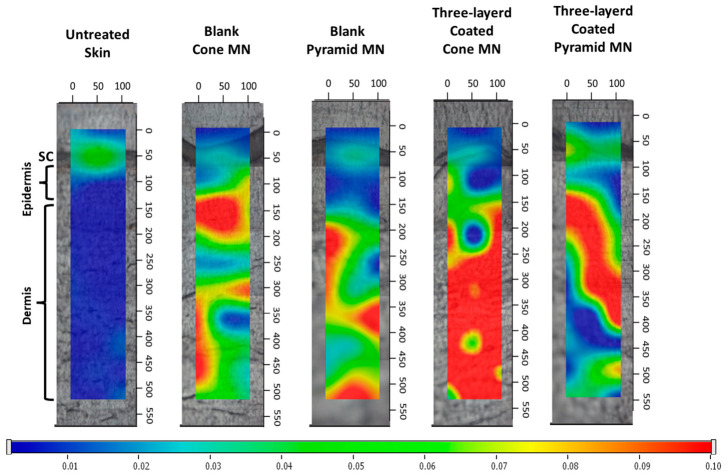
Raman correlation maps of excised human full-thickness skin under various experimental conditions. Untreated control skin; skin treated with a blank conical microneedle; skin treated with a blank pyramidal microneedle; skin treated with a three-layer-coated conical microneedle; skin treated with a three-layer-coated pyramidal microneedle. Warmer colors indicate higher levels of formulation deposition within the cutaneous layers. SC: Stratum Corneum.

Raman mapping demonstrated that both conical and pyramidal microneedle geometries successfully delivered the lidocaine-loaded hydrogel to the deeper layers of the skin, as indicated by higher color intensity reflecting the presence of the lidocaine formulation [70]. However, conical microneedles produced a more intense drug signal at greater penetration depth compared with the pyramidal design, revealing more efficient delivery. This improved performance is consistent with their enhanced mechanical robustness, which preserves sharper needle tips and minimizes height reduction under mechanical stress (Figure 13).

These findings highlight the critical role of microneedle design and processing parameters in governing insertion behavior and delivery performance, with a need for further quantitative analysis to optimize transdermal delivery outcomes.

## 4. Conclusions

This study demonstrates the feasibility of LCD-based vat photopolymerization as a reliable high-resolution additive manufacturing technique for fabricating microneedle arrays. This approach is scalable and highly customizable for dermal and transdermal drug delivery applications. Microneedle geometry, hydrogel formulation, and coating method were identified as key factors influencing mechanical properties, coating homogeneity, drug delivery performance, and skin permeation. Conical microneedles showed higher mechanical strength and enabled deeper and more effective intradermal deposition of lidocaine, whereas pyramidal microneedles exhibited higher drug loading capacity, likely due to their increased surface area. Regarding coating parameters, increased polymer content enabled the formation of uniform multilayer coatings, contributing to effective skin permeation in an ex vivo human skin model.

Further work should address long-term physical and chemical stability under varying environmental conditions and the effects of sterilization on material and coating integrity. Benchmarking against commercially available delivery systems is essential to position the performance of the proposed platform in clinically relevant contexts. Considering that the dose required for effective local anesthesia is higher than that delivered by the current prototype, clinically relevant application will require appropriate scale-up strategies, such as increasing array surface area, optimizing coating thickness, or implementing parallel array fabrication. These factors are critical for ensuring reproducibility, safety, and reliable performance in potential clinical applications. In vivo studies will also be essential for elucidating pharmacokinetic profiles and validating clinical performance.

Overall, the combination of LCD-based 3D printing with customizable hydrogel coatings may represent a versatile platform for developing patient-specific microneedle systems. Future studies may explore a broader range of therapeutic agents, including biologics (e.g., peptides and vaccines) and small-molecule drugs, to further assess the versatility and generalizability of this drug delivery system.

## Figures and Tables

**Table 1:** Comparison of 3D printing methods for microneedle fabrication.

Methods	Layer Thickness (μm)	Printer Cost ($)	Printing Speed (mm/h)	MN Suitability	Applicability	Citation
LCD	50–100	200–2300	20–80	High: Sharp tips (40–60 μm), biocompatible resins	Ideal: High resolution, low cost, rapid prototyping	[20,21,22,23]
SLA	10–400 (most common: 50)	3500–100,000	14–36	High: Precise MN geometries	Viable but costlier and slower than LCD	[20,21,22,24]
DLP	25–100 (most common: 50)	2000–4000	20–140	High: Smooth surfaces, fast	Compared to LCD, slightly costlier	[20,21,22,24,25]
2PP	0.1–5	400,000+	Hours/array	Very high: Ultra-fine tips	Impractical due to cost and slow speed	[21,26,27]
FDM	50–300 (most common: 200)	200–200,000	50–150	Low: Coarse resolution	Unsuitable for sharp MN tips	[20,21,28,29]
CLIP	50–100	64,000–162,500	10–25× faster than SLA	Moderate: Fast but lower resolution	Viable but expensive, less precise	[20,21,30]
PBF	20–150 (most common: 100)	24,000–650,000	20–60	Low–Moderate: Limited resolution and surface roughness	Better for metals/functional parts, not ideal for fine MN tips	[21,29,31,32]

Abbreviations: Liquid crystal display (LCD), stereolithography (SLA), digital light processing (DLP), two-photon polymerization (2PP), fused deposition modeling (FDM), continuous liquid interface production (CLIP), powder bed fusion (PBF).

**Table 2:** Composition of the hydrogels used in the tests (% *w*/*w*).

Component	Hydrogel 1	Hydrogel 2	Blank Hydrogel 1	Blank Hydrogel 2
Lidocaine	2	2	-	-
Ethanol 96%	50	50	50	50
Glycerol 85%	10	10	10	10
Carbopol^®^ EZ-3	1	0.6	1	0.6
Triisopropanolamine solution 40%	-	-	qu. s.	qu. s.
Deionized water	up to 100	up to 100	up to 100	up to 100

**Table 3:** pH values of lidocaine-loaded hydrogels and their corresponding unloaded controls, formulated with varying concentrations of Carbopol^®^ EZ-3.

Formulation	pH ± SD
Hydrogel 1	7.27 ± 0.02
Blank Hydrogel 1	5.65 ± 0.03
Hydrogel 2	7.57 ± 0.02
Blank Hydrogel 2	5.35 ± 0.03

**Table 4:** Parameters characterizing spreadability and adhesive properties.

Formulations	Firmness (mN)	Adhesion Force (mN)	Work to Spread (mN·s)	Adhesiveness (mN·s)
Hydrogel 1	127.07 ± 5.55	−55.97 ± 5.22	704.22 ± 58.99	−692.58 ± 37.45
Blank Hydrogel 1	109.76 ± 6.67	−51.29 ± 9.86	734.78 ± 121.52	−586.45 ± 101.67
Hydrogel 2	95.58 ± 8.99	−49.54 ± 6.04	643.55 ± 120.50	−590.56 ± 111.21
Blank Hydrogel 2	62.86 ± 3.00	−35.21 ± 2.00	485.26 ± 14.00	−481.99 ± 20.00

**Table 5:** Apparent viscosity of the formulations at a shear rate of 50 s^−1^.

Formulation	Viscosity (mPa·s) ± SD
Hydrogel 1	6855 ± 128
Hydrogel 2	4118 ± 17
Blank Hydrogel 1	6082 ± 259
Blank Hydrogel 2	3220 ± 28

**Table 6:** Drug content per individual MNA. Data are expressed as mean ± SD (*n* = 3).

Microneedle Shape	Drug Content (µg)	Total Lateral Surface of MNA (µm^2^)
Cone	216.47 ± 6.10	1,166,389
Pyramid	236.47 ± 11.74	1,484,318

## Data Availability

The data presented in this study are available on request from the corresponding author. The data is not publicly available due to ongoing research activities.

## Supplementary Materials

The following supporting information can be downloaded at: https://www.mdpi.com/article/10.3390/pharmaceutics18050518/s1, Video S1: Dynamic visualization of microneedle-pierced skin, showing fluorescence distribution along the insertion pathway, penetration depth, and hydrogel localization.

## Abbreviations

The following abbreviations are used in this manuscript:

2PP	Two-Photon Polymerization
ANOVA	Analysis of Variance
APC	Article Processing Charge
CAD	Computer-Aided Design
CCD	Charge-Coupled Device
CLIP	Continuous Liquid Interface Production
DLP	Digital Light Processing
FDM	Fused Deposition Modeling
G′	Storage Modulus
G″	Loss Modulus
HCPL	High-Contrast Plan Fluotar
HPLC	High-Performance Liquid Chromatography
IVRT	In Vitro Release Testing
LCD	Liquid Crystal Display
LVER	Linear Viscoelastic Region
MCR	Modular Compact Rheometer
MN	Microneedle
MNAs	Microneedle Arrays
PBS	Phosphate-Buffered Saline
PBF	Powder Bed Fusion
SC	Stratum Corneum
SLA	Stereolithography
STL	Standard Tessellation Language
UV	Ultraviolet
VPP	Vat Photopolymerization

## References

[1] Richard C., Cassel S., Blanzat M. (2021). Vesicular systems for dermal and transdermal drug delivery. RSC Adv..

[2] Raina N., Rani R., Thakur V.K., Gupta M. (2023). New Insights in Topical Drug Delivery for Skin Disorders: From a Nanotechnological Perspective. ACS Omega.

[3] Owsley A., Misra R., Awe A., Ma J., Verma G., Velaoras A.T., Glenn P., Frasier K. (2025). Skin Based Delivery Systems for Therapeutic Molecules: Advancing Dermatological Treatments through Innovative Drug Delivery Technologies. Dermis.

[4] Prausnitz M.R., Langer R. (2008). Transdermal drug delivery. Nat. Biotechnol..

[5] Ita K. (2015). Transdermal Delivery of Drugs with Microneedles-Potential and Challenges. Pharmaceutics.

[6] Nguyen H.X., Banga A.K. (2025). Advanced transdermal drug delivery system: A comprehensive review of microneedle technologies, novel designs, diverse applications, and critical challenges. Int. J. Pharm..

[7] Olatunji O., Das D.B., Garland M.J., Belaid L., Donnelly R.F. (2013). Influence of array interspacing on the force required for successful microneedle skin penetration: Theoretical and practical approaches. J. Pharm. Sci..

[8] Bian S., Chen J., Chen R., Feng S., Ming Z. (2025). Enhanced Transdermal Delivery of Lidocaine Hydrochloride via Dissolvable Microneedles (LH-DMNs) for Rapid Local Anesthesia. Biosensors.

[9] Zafar S., Rana S.J., Hamza M., Hussain A., Abbas N., Ghori M.U., Arshad M.S. (2025). Advancements in transdermal drug delivery using microneedles: Technological and material perspective. Discov. Pharm. Sci..

[10] Tang J.-F., Lin K.-W., Lin T.-H., Lin W.-C. (2025). Pioneering techniques for achieving high-resolution, ultrasmooth surfaces via LCD 3D printing technology. Addit. Manuf..

[11] Dave H.K., Patel S.T., Dave H.K., Davim J.P. (2021). Introduction to Fused Deposition Modeling Based 3D Printing Process. Fused Deposition Modeling Based 3D Printing.

[12] Shafique H., Karamzadeh V., Kim G., Shen M.L., Morocz Y., Sohrabi-Kashani A., Juncker D. (2024). High-resolution low-cost LCD 3D printing for microfluidics and organ-on-a-chip devices. Lab Chip.

[13] Krieger K.J., Bertollo N., Dangol M., Sheridan J.T., Lowery M.M., O’Cearbhaill E.D. (2019). Simple and customizable method for fabrication of high-aspect ratio microneedle molds using low-cost 3D printing. Microsyst. Nanoeng..

[14] Pere C.P.P., Economidou S.N., Lall G., Ziraud C., Boateng J.S., Alexander B.D., Lamprou D.A., Douroumis D. (2018). 3D printed microneedles for insulin skin delivery. Int. J. Pharm..

[15] Lim S.H., Ng J.Y., Kang L. (2017). Three-dimensional printing of a microneedle array on personalized curved surfaces for dual-pronged treatment of trigger finger. Biofabrication.

[16] Xu X., Awad A., Robles-Martinez P., Gaisford S., Goyanes A., Basit A.W. (2021). Vat photopolymerization 3D printing for advanced drug delivery and medical device applications. J. Control. Release.

[17] Rad Z.F., Prewett P.D., Davies G.J. (2021). High-resolution two-photon polymerization: The most versatile technique for the fabrication of microneedle arrays. Microsyst. Nanoeng..

[18] Dabbagh S.R., Sarabi M.R., Rahbarghazi R., Sokullu E., Yetisen A.K., Tasoglu S. (2021). 3D-printed microneedles in biomedical applications. iScience.

[19] Economidou S.N., Lamprou D.A., Douroumis D. (2018). 3D printing applications for transdermal drug delivery. Int. J. Pharm..

[20] Detamornrat U., McAlister E., Hutton A.R.J., Larrañeta E., Donnelly R.F. (2022). The Role of 3D Printing Technology in Microengineering of Microneedles. Small.

[21] Sirbubalo M., Tucak A., Muhamedagic K., Hindija L., Rahić O., Hadžiabdić J., Cekic A., Begic-Hajdarevic D., Husic M.C., Dervišević A. (2021). 3D Printing—A “Touch-Button” Approach to Manufacture Microneedles for Transdermal Drug Delivery. Pharmaceutics.

[22] LCD vs. DLP vs. SLA: Which Resin 3D Printer Is Best? Phrozen. https://phrozen3d.com/en/blogs/guides/sla-vs-dlp-vs-lcd-which-resin-3d-printer-is-the-best-for-you.

[23] Compare Formlabs SLA 3D Printers’ Tech Specs, Formlabs. https://formlabs.com/global/3d-printers/resin/tech-specs/.

[24] Ravi P., Patel P., Banerjee S. (2023). Stereolithography (SLA) in Pharmaceuticals. Additive Manufacturing in Pharmaceuticals.

[25] (2023). DLP vs. LCD 3D Printer: The Main Differences, All3DP. https://all3dp.com/2/lcd-vs-dlp-3d-printing-technologies-compared/.

[26] Wu S., Serbin J., Gu M. (2006). Two-photon polymerisation for three-dimensional micro-fabrication. J. Photochem. Photobiol. A Chem..

[27] Baker-Sediako R.D., Richter B., Blaicher M., Thiel M., Hermatschweiler M. (2023). Industrial perspectives for personalized microneedles. Beilstein J. Nanotechnol..

[28] Awad A., Trenfield S.J., Goyanes A., Gaisford S., Basit A.W. (2018). Reshaping drug development using 3D printing. Drug Discov. Today.

[29] (2025). r3-D2, SLA Printing and Resin 3D Printing: A Complete Guide to Stereolithography Technology, 3D Mag. https://www.3dmag.com/3d-wikipedia/sla-printing-resin-3d-printing-stereolithography-guide/.

[30] Poole L. (2025). CLIP 3D Printing Manufacturing: Complete Guide to Continuous Liquid Interface Production Technology—ModernTechMech.com. https://www.moderntechmech.com/clip-3d-printing-manufacturing/.

[31] Kumar S., Malviya R., Sridhar S.B., Wadhwa T., Hani U., Talath S., Warsi M.H. (2025). Powder bed fusion 3D printing for drug delivery and healthcare applications. Ann. 3D Print. Med..

[32] (2024). The Complete Guide to SLS 3D Printing, All3DP Pro. https://all3dp.com/1/sls-3d-printing-the-ultimate-guide/.

[33] Li Q.Y., Zhang J.N., Chen B.Z., Wang Q.L., Guo X.D. (2017). A solid polymer microneedle patch pretreatment enhances the permeation of drug molecules into the skin. RSC Adv..

[34] Sartawi Z., Blackshields C., Faisal W. (2022). Dissolving microneedles: Applications and growing therapeutic potential. J. Control. Release.

[35] Kim J., Jeong J., Jo J.K., So H. (2025). Hollow microneedles as a flexible dosing control solution for transdermal drug delivery. Mater. Today Bio.

[36] Ingrole R.S.J., Gill H.S. (2019). Microneedle Coating Methods: A Review with a Perspective. J. Pharmacol. Exp. Ther..

[37] Mohite P., Puri A., Munde S., Ade N., Kumar A., Jantrawut P., Singh S., Chittasupho C. (2024). Hydrogel-Forming Microneedles in the Management of Dermal Disorders Through a Non-Invasive Process: A Review. Gels.

[38] Haj-Ahmad R., Khan H., Arshad M.S., Rasekh M., Hussain A., Walsh S., Li X., Chang M.-W., Ahmad Z. (2015). Microneedle Coating Techniques for Transdermal Drug Delivery. Pharmaceutics.

[39] Gill H.S., Prausnitz M.R. (2007). Coated microneedles for transdermal delivery. J. Control. Release.

[40] Liang L., Chen Y., Zhang B.L., Zhang X.P., Liu J.L., Shen C.B., Cui Y., Guo X.D. (2020). Optimization of dip-coating methods for the fabrication of coated microneedles for drug delivery. J. Drug Deliv. Sci. Technol..

[41] Kim S.-J., Shin J.-H., Noh J.-Y., Song C.-S., Kim Y.-C. (2016). Development of the novel coating formulations for skin vaccination using stainless steel microneedle. Drug Deliv. Transl. Res..

[42] Crasta A., Painginkar T., Sreedevi A., Pawar S.D., Sathyanarayana M.B., Vasantharaju S.G., Osmani R.A.M., Ravi G. (2025). Transdermal drug delivery system: A comprehensive review of innovative strategies, applications, and regulatory perspectives. OpenNano.

[43] Qiao X., Li L. (2024). A novel lidocaine-chitosan-barium titanate microemulsion gel for prolonged local anesthesia: An in vitro study. Arch. Biol. Sci..

[44] Larrañeta E., Lutton R.E.M., Woolfson A.D., Donnelly R.F. (2016). Microneedle arrays as transdermal and intradermal drug delivery systems: Materials science, manufacture and commercial development. Mater. Sci. Eng. R Rep..

[45] Bahar E., Yoon H., Anesthetic A.L. (2021). Its Adverse Effects and Management. Medicina.

[46] Filho D., Guerrero M., Pariguana M., Marican A., Durán-Lara E.F. (2023). Hydrogel-Based Microneedle as a Drug Delivery System. Pharmaceutics.

[47] Loh J.M., Lim Y.J.L., Tay J.T., Cheng H.M., Tey H.L., Liang K. (2024). Design and fabrication of customizable microneedles enabled by 3D printing for biomedical applications. Bioact. Mater..

[48] Aldawood F.K., Andar A., Desai S. (2021). A Comprehensive Review of Microneedles: Types, Materials, Processes, Characterizations and Applications. Polymers.

[49] Kordyl O., Styrna Z., Wojtyłko M., Dlugaszewska J., Kaminska D., Murias M., Mlynarczyk D.T., Jadach B., Skotnicka A., Michniak-Kohn B. (2025). Optimization of LCD-Based 3D Printing for the Development of Clotrimazole-Coated Microneedle Systems. Materials.

[50] Larrañeta E., Moore J., Vicente-Pérez E.M., González-Vázquez P., Lutton R., Woolfson A.D., Donnelly R.F. (2014). A proposed model membrane and test method for microneedle insertion studies. Int. J. Pharm..

[51] Anjani Q.K., Nainggolan A.D.C., Li H., Miatmoko A., Larrañeta E., Donnelly R.F. (2024). Parafilm^®^ M and Strat-M^®^ as skin simulants in in vitro permeation of dissolving microarray patches loaded with proteins. Int. J. Pharm..

[52] Vora L.K., Vavia P.R., Larrañeta E., Bell S.E.J., Donnelly R.F. (2018). Novel nanosuspension-based dissolving microneedle arrays for transdermal delivery of a hydrophobic drug. J. Interdiscip. Nanomed..

[53] Alrimawi B.H., Lee J.Y., Ng K.W., Goh C.F. (2024). In vitro evaluation of microneedle strength: A comparison of test configurations and experimental insights. RSC Pharm..

[54] Chanabodeechalermrung B., Chaiwarit T., Udomsom S., Rachtanapun P., Piboon P., Jantrawut P. (2024). Determination of vat-photopolymerization parameters for microneedles fabrication and characterization of HPMC/PVP K90 dissolving microneedles utilizing 3D-printed mold. Sci. Rep..

[55] Zsikó S., Cutcher K., Kovács A., Budai-Szűcs M., Gácsi A., Baki G., Csányi E., Berkó S. (2019). Nanostructured Lipid Carrier Gel for the Dermal Application of Lidocaine: Comparison of Skin Penetration Testing Methods. Pharmaceutics.

[56] DeMuth P.C., Min Y., Huang B., Kramer J.A., Miller A.D., Barouch D.H., Hammond P.T., Irvine D.J. (2013). Polymer multilayer tattooing for enhanced DNA vaccination. Nat. Mater..

[57] Kovács A., Falusi F., Gácsi A., Budai-Szűcs M., Csányi E., Veréb Z., Monostori T., Csóka I., Berkó S. (2024). Formulation and investigation of hydrogels containing an increased level of diclofenac sodium using risk assessment tools. Eur. J. Pharm. Sci..

[58] Tfayli A., Piot O., Pitre F., Manfait M. (2007). Follow-up of drug permeation through excised human skin with confocal Raman microspectroscopy. Eur. Biophys. J..

[59] Kiss E.L., Berkó S., Gácsi A., Kovács A., Katona G., Soós J., Csányi E., Gróf I., Harazin A., Deli M.A. (2019). Design and Optimization of Nanostructured Lipid Carrier Containing Dexamethasone for Ophthalmic Use. Pharmaceutics.

[60] Migliozzi S., Meridiano G., Angeli P., Mazzei L. (2020). Investigation of the swollen state of Carbopol molecules in non-aqueous solvents through rheological characterization. Soft Matter.

[61] Stancu A.I., Oprea E., Dițu L.M., Ficai A., Ilie C.-I., Badea I.A., Buleandra M., Brîncoveanu O., Ghica M.V., Avram I. (2024). Development, Optimization, and Evaluation of New Gel Formulations with Cyclodextrin Complexes and Volatile Oils with Antimicrobial Activity. Gels.

[62] Wojtyłko M., Nowicka A.B., Froelich A., Szybowicz M., Banaszek T., Tomczak D., Kuczko W., Wichniarek R., Budnik I., Jadach B. (2025). Characteristics of Hydrogels as a Coating for Microneedle Transdermal Delivery Systems with Agomelatine. Molecules.

[63] Tabernero A., Guastaferro M., González-Garcinuño Á., Misol A., Baldino L., Cardea S., del Valle E.M., Reverchon E. (2022). The viscoelastic behavior of the precursor hydrogels can modify aerogel properties. J. Supercrit. Fluids.

[64] Kavčič H., Jug U., Mavri J., Umek N. (2023). Antioxidant activity of lidocaine, bupivacaine, and ropivacaine in aqueous and lipophilic environments: An experimental and computational study. Front. Chem..

[65] Sayhan H., Beyaz S.G., Çeliktaş A., Georgiev V., Pavlov A. (2017). The Local Anesthetic and Pain Relief Activity of Alkaloids. Alkaloids—Alternatives in Synthesis, Modification and Application.

[66] Whizar-Lugo V.M., Hernández-Cortez E. (2020). Topics in Local Anesthetics.

[67] Jimenez-Kairuz A., Allemandi D., Manzo R.H. (2002). Mechanism of Lidocaine Release from Carbomer–Lidocaine hydrogels. J. Pharm. Sci..

[68] Miguel A.A. (2018). Mass Transfer Within Complex Media. Reverse Engineering: From Usage Property to Material. Ph.D. Thesis.

[69] George A., Limbachiya V., Shrivastav P.S. (2025). Current status and role of carbopols in oral, nasal. transdermal, topical and ophthalmic drug delivery systems. Next Mater..

[70] Hasanpour F., Budai-Szűcs M., Kovács A., Ambrus R., Jójárt-Laczkovich O., Cseh M., Geretovszky Z., Ayaydin F., Berkó S. (2024). Improvement of lidocaine skin permeation by using passive and active enhancer methods. Int. J. Pharm..

